# Mechanisms underlying the lack of endogenous processing and CLIP-mediated binding of the invariant chain by HLA-DP^84Gly^

**DOI:** 10.1038/s41598-018-22931-4

**Published:** 2018-03-19

**Authors:** Mark Anczurowski, Yuki Yamashita, Munehide Nakatsugawa, Toshiki Ochi, Yuki Kagoya, Tingxi Guo, Chung-Hsi Wang, Muhammed A. Rahman, Kayoko Saso, Marcus O. Butler, Naoto Hirano

**Affiliations:** 1 Tumor Immunotherapy Program, Campbell Family Institute for Breast Cancer Research, Campbell Family Cancer Research Institute, Princess Margaret Cancer Centre, University Health Network, Toronto, Ontario M5G 2M9 Canada; 2 Department of Immunology, University of Toronto, Toronto, Ontario M5S 1A8 Canada; 3 Department of Medicine, University of Toronto, Toronto, Ontario M5S 1A8 Canada

## Abstract

While the principles of classical antigen presentation via MHC class II are well-established, the mechanisms for the many routes of cross-presentation by which endogenous antigens become associated with class II molecules are not fully understood. We have recently demonstrated that the single amino acid polymorphism HLA-DPβ^84Gly^ (DP^84Gly^) is critical to abrogate class II invariant chain associated peptide (CLIP) region-mediated binding of invariant chain (Ii) to DP, allowing endoplasmic reticulum (ER)-resident endogenous antigens to constitutively associate with DP^84Gly^ such as DP4. In this study, we demonstrate that both the CLIP and N-terminal non-CLIP Ii regions cooperatively generate an Ii conformation that cannot associate with DP^84Gly^ via the CLIP region. We also demonstrate the ability of DP4 to efficiently process and present antigens encoded in place of CLIP in a chimeric Ii, regardless of wild type Ii and HLA-DM expression. These data highlight the complex interplay between DP polymorphisms and the multiple Ii regions that cooperatively regulate this association, ultimately controlling the presentation of endogenous antigens on DP molecules. These results may also offer a mechanistic explanation for recent studies identifying the differential effects between DP^84Gly^ and DP^84Asp^ as clinically relevant in human disease.

## Introduction

The foundations of classical class II presentation of exogenous peptides are well established. MHC class II molecules synthesized in the endoplasmic reticulum (ER) rapidly associate with a homotrimeric assembly of the non-polymorphic, type II transmembrane glycoprotein invariant chain (Ii) via its class II-associated invariant chain peptide (CLIP) region, as well as other non-CLIP regions^1^. The role of Ii in class II biology is three-fold and integral to normal class II functioning. First, Ii is able to block the class II cleft to prevent the binding of endogenously derived peptides in the ER^2^. Second, Ii facilitates the formation of nonamers with class II via its CLIP region, facilitating efficient class II egress from the ER and progression to the endocytic pathway^3,4^. Third, Ii acts as a chaperone for class II, not only aiding in the folding of class II shortly after synthesis, but also shuttling class II through the endocytic pathway to the MIIC compartment where it is degraded, leaving only the CLIP peptide bound to the class II cleft^5–8^. CLIP is then removed by the activity of HLA-DM to allow exogenously derived antigens to be loaded onto the now-receptive class II molecule^8^.

In addition to the presentation of exogenous peptides, class II molecules can also present antigens derived from endogenous sources. The appearance of endogenous antigens bound to class II has previously been explained by autophagy and recycling pathway-centered theories, for which roles have been convincingly demonstrated for both macroautophagy^9–11^ and chaperone-mediated autophagy^12–14^. Other studies have also identified components of the class I processing pathway as involved in cross-presentation on class II molecules. In particular, both the proteasome and the transporter associated with antigen processing (TAP) have documented roles in this process^15^. The association of peptides generated through such a pathway is conceptually straightforward to visualize in the absence of Ii; such antigens could simply bind to class II unimpeded in the ER before exogenous antigens would become available later in the endocytic pathway. The mechanisms that allow a parallel process to occur in Ii-positive cells had, until recently, remained unknown.

Recent work from our laboratory has uncovered a pathway by which endogenous peptides can associate with a subgroup of class II alleles regardless of Ii expression^16^. HLA-DP alleles can be divided into two categories based on the amino acid encoded at position 84 of the DPβ chain, virtually always either Gly (DP^84Gly^) or Asp (DP^84Asp^). We demonstrated that DP^84Gly^ is unable to bind the CLIP region of Ii and is unable to produce nor display CLIP derived from full-length, endogenous Ii on the cell surface. Consequently, the association of intracellular peptides with DP^84Gly^ is not blocked by Ii, presumably in the ER, resulting in constitutive presentation of both endogenous and exogenous antigens on DP^84Gly^. Importantly, Ii is still capable of binding to DP^84Gly^ via non-CLIP regions regardless of this polymorphism and facilitating its progress through the endocytic pathway^16^. DP^84Gly^ also depends on components of the class I processing pathway, such as the proteasome and TAP, for the presentation of endogenous peptides revealing the involvement of key class I-associated machinery. Strikingly, this phenotype depends on the single amino acid polymorphism at position 84 of the DPβ chain. Although the four polymorphic amino acids at positons 84–87 of the DPβ chain are in complete linkage disequilibrium, generally as either DPβ^84GGPM87^ or DPβ^84DEAV87^, substitution of only Asp^84^ to Gly^84^ or vice-versa is required and sufficient to completely reverse these phenomena^17^. Given the genetic linkage of these amino acids, we used the four amino acid substitution mutant DP4^84DEAV87^ throughout the current study to represent DP^84Asp^-type DP alleles, and henceforth will refer to the DP alleles encoding these polymorphisms as DP^84GGPM87^ and DP^84DEAV87^ for the sake of clarity.

Aside from our previous investigations into the nature of the DPβ^84^ polymorphism on DP binding to Ii, little is known regarding the nature of this inhibitory interaction from the Ii perspective. The region(s) of Ii that mediate the inhibition of CLIP-mediated binding to DP^84GGPM87^ have yet to be explored, and the relative contributions of both CLIP and non-CLIP interactions with DP in determining the final nature of the association between these two molecules remain understudied. The observation that CLIP-mediated DP/Ii association directly influences the peptide repertoire presented by class II, in addition to several studies suggesting the use of Ii as a vehicle to deliver specific antigens to the class II processing pathway, highlights the importance of studying the nature of the class II/Ii interaction. Specifically, it is necessary to determine under what conditions CLIP-mediated DP/Ii interactions exist, and how altering the Ii amino acid sequence may modulate its binding to, and ultimately transport though, the endocytic pathway.

In this study, we delineate the regions of Ii that contribute to the lack of CLIP-mediated binding of Ii to DP^84GGPM87^. We utilize a chimeric Ii construct encoding the known DP4-restricted peptide MAGE-A3_243–258_ in place of CLIP to demonstrate that a different, yet still compatible CLIP region sequence can restore canonical multimer formation between Ii and DP4. We also demonstrate the highly efficient presentation of MAGE-A3_243–258_ peptide from this construct to the cell surface on both types of HLA-DP alleles, as has been demonstrated with other class II molecules. We characterize this presentation as robust on DP4 regardless of the presence of both wild-type Ii and HLA-DM, and suggest that the loading of MAGE-A3 antigen derived from chimeric Ii proceeds similarly to the loading of peptides derived from full-length endogenous MAGE-A3. Furthermore, we study the influence of non-CLIP region-mediated factors in the lack of CLIP-mediated Ii/DP association. By utilizing truncated Ii mutants, we reveal the involvement of N-terminal non-CLIP Ii region(s) in the absence CLIP-mediated Ii association with DP4. Thus both the CLIP and N-terminal non-CLIP regions of Ii jointly contribute to the final Ii conformation, determining the presence or absence of CLIP-mediated binding to DP, and ultimately controlling the repertoire of peptides available to be presented on HLA-DP.

## Results

### HLA-DM, which removes CLIP from DP^84DEAV87^, does not affect the lack of CLIP displayed by DP^84GGPM87^

**Figure 1:**
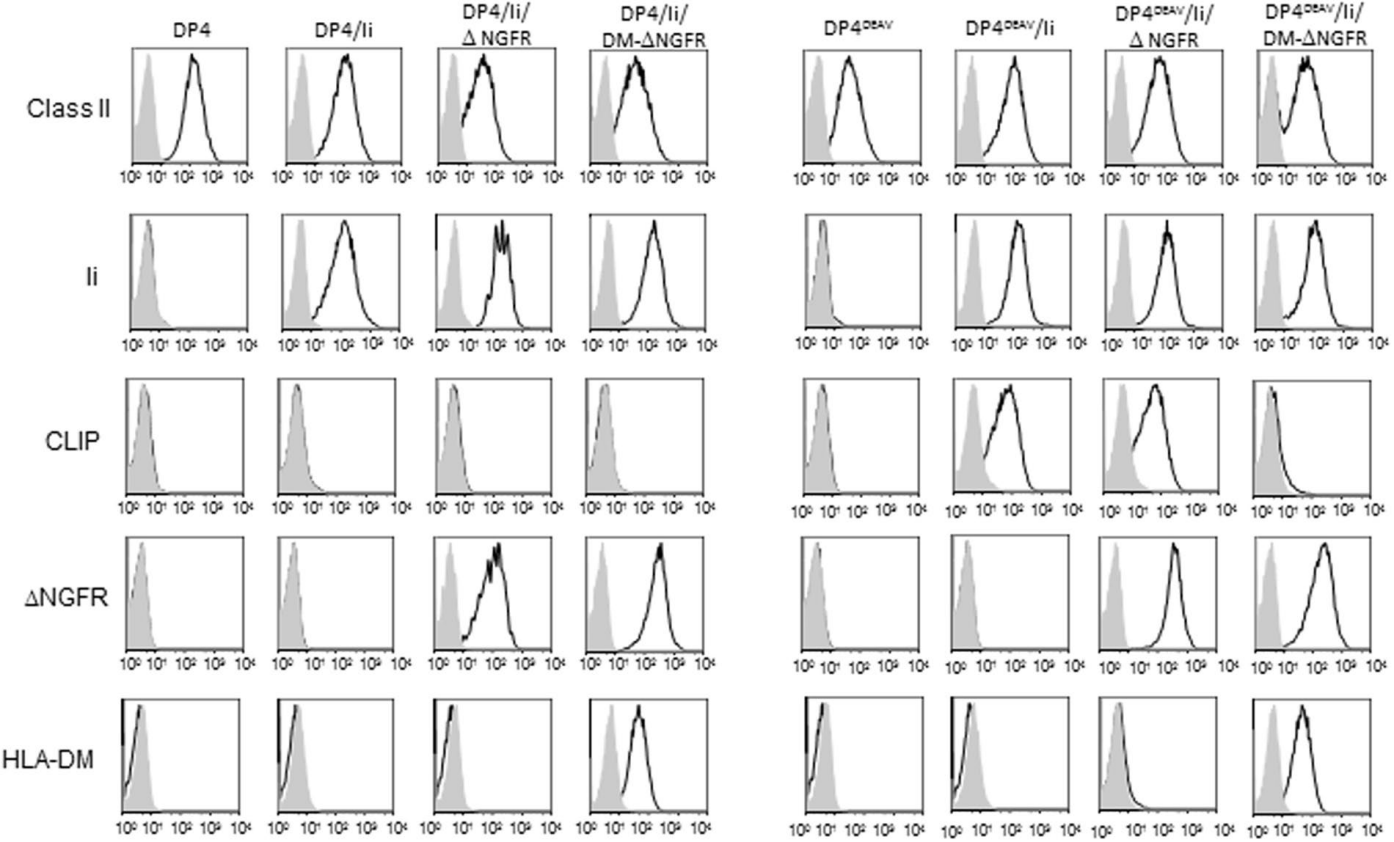
CLIP is not displayed by HLA-DP molecules encoding the DP^84GGPM87^ phenotype regardless of HLA-DM expression on K562 cells. Surface class II, CLIP and ΔNGFR expression, along with intracellular Ii and HLA-DM expression, were analyzed on K562 transfected cells by flow cytometry following staining with specific mAbs. K562 cells were retrovirally transduced with DPA1*01:03 (DPA1) and either wild-type or mutated DPB1*04:01 (DPB4), with substitution mutations of the 84–87 region of the DPβ chain made as indicated to generate DP4 and DP4^84DEAV87^ respectively.

HLA-DP^84GGPM87^do not contain CLIP derived from full length Ii on the cell surface^16^. To confirm these findings and assess the effect of HLA-DM on the lack of CLIP observed, we generated a series of K562 transfectants stably expressing Ii and either DP4 or DP4^84DEAV87^ in the presence or absence of HLA-DM in a similar manner as previously published^16^. K562 cells are deficient in class II, Ii, and HLA-DM expression but possess the remaining antigen processing and presentation machinery such that transfection of class I or II results in functional antigen presentation^18–20^. As expected, K562 cells expressing DP4^84DEAV87^ demonstrated robust CLIP expression on the cell surface, which could be strongly abrogated by the presence of HLA-DM (Fig. 1). However, wild-type DP4-expressing K562 cells did not contain CLIP regardless of HLA-DM expression. To generate the indicated DP heterodimers, the DPβ chain was transfected in conjunction with DPA1*01:03 (DPA1) in all cases. Importantly, the use of DPA1*02:01 (DPA2) in lieu of DPA1 does not affect these results^16^. To validate these results, similar findings were obtained using HEK293 cells that were also deficient in endogenous class II, Ii, and HLA-DM expression (Supplementary Fig. 1a). Together, these results indicate that DM does not affect the lack of CLIP observed on the cell surface, regardless of the backbone cell line or *DPA* gene utilized.

### Synthetic CLIP peptide can bind to DP4 and DP4^84DEAV87^ similarly

**Figure 2:**
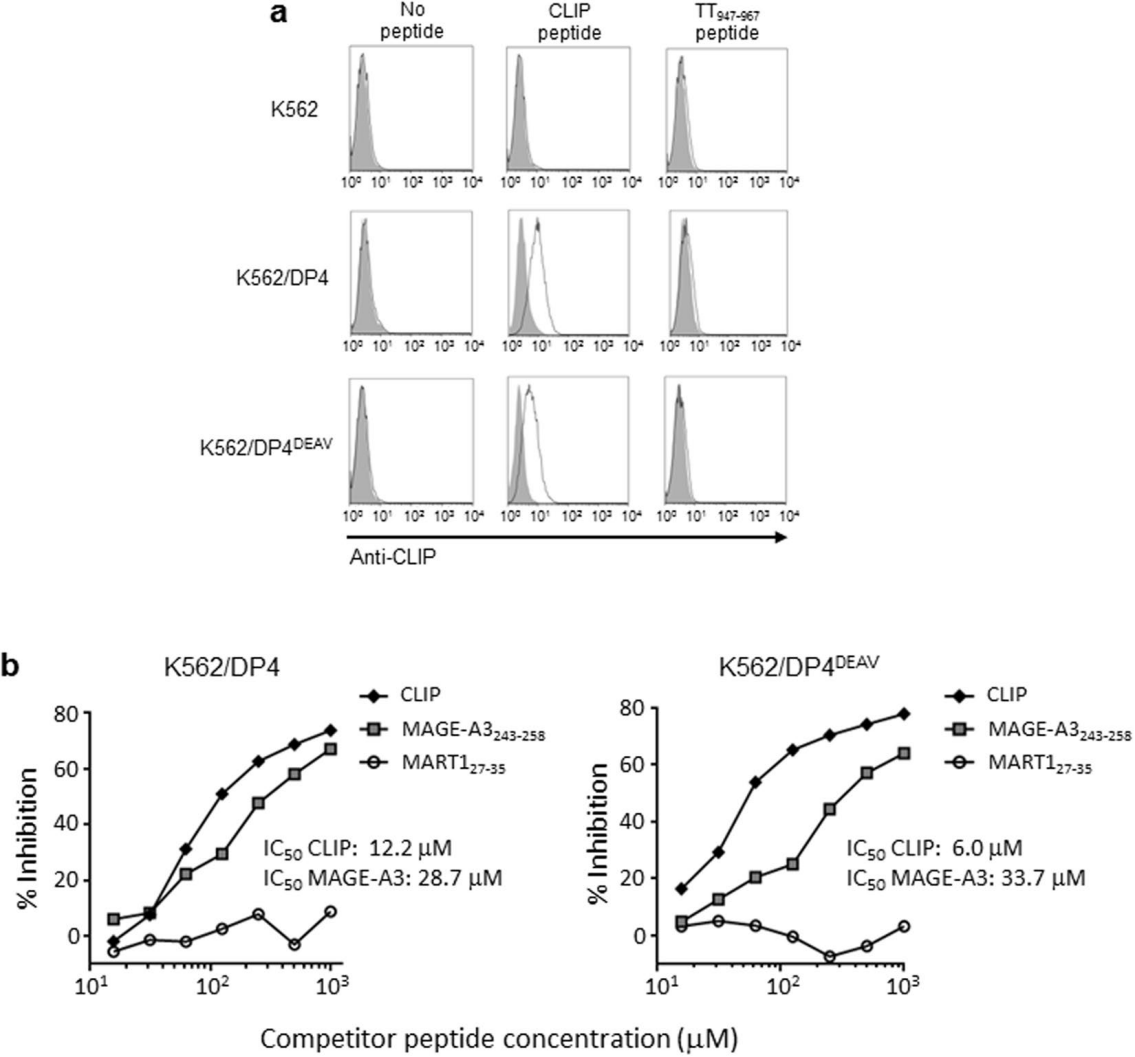
The CLIP peptide binds to both DP4 and DP4^84DEAV87^ with greater binding strength than the known DP4-restricted MAGE-A3_243–258_ peptide. (**a**) Parental K562 or K562 cells expressing one of DP4 or DP4^84DEAV87^ were pulsed with 100 μM of CLIP, TT_947–967_, or an equivalent volume of DMSO for 18 hrs. Surface CLIP expression was then analyzed by flow cytometry on the indicated K562 transfected cells following staining with CLIP-specific mAb. (**b**) A K562 cell-based competitive binding assay was performed using K562-based transfected cells encoding either DP4 or DP4^84DEAV87^, pulsed with graded concentrations of CLIP, MAGE-A3_243–258_, or MART1_27–35_ peptide in the presence of 2 μM biotin-conjugated reference peptide, HIV ENV_31–45_. Cells were then stained by phycoerythrin-conjugated streptavidin and analyzed by flow cytometry. Percent inhibition of binding by CLIP peptide was calculated by Mean Fluorescence Intensity (MFI) using the following formula: %inhibition = [1 − (MFI with CLIP peptide/MFI without CLIP peptide)] × 100.

Our previous observations that DP^84GGPM87^ does not bind Ii via the CLIP region nor contain the CLIP peptide on the cell surface, prompted us to hypothesize that the CLIP peptide itself does not associate with these DPs or at least far less efficiently compared with DP^84DEAV87^. Thus, we pulsed chemically synthesized CLIP peptide on K562 cells expressing either DP4 or DP4^84DEAV87^ and assessed its binding by flow cytometry. Surprisingly, the CLIP peptide did bind comparably to both DP4 and DP4^84DEAV87^ (Fig. 2a). Furthermore, we performed a competitive binding assay using these two K562 cell lines to compare the binding ability of both CLIP peptide and the known DP4-restricted peptide MAGE-A3_243–258_ to DP4 and DP4^84DEAV87^ ^18^. Both the CLIP and MAGE-A3_243–258_ peptides bound to DP4 and DP4^84DEAV87^ with comparable IC_50_ values, although surprisingly, CLIP had a superior binding capacity for both DP molecules compared to MAGE-A3_243–258_ (Fig. 2b). We further validated the binding of synthetic CLIP peptide to both DP alleles using DP4 or DP4^84DEAV87^ -transduced HEK293 cells in flow cytometry analysis as described above (Supplementary Fig. 1b). These results suggest that the CLIP peptide sequence itself is not incompatible with the cleft of DP^84GGPM87^. Rather, it is conceivable that either the total conformation of Ii is not amenable to CLIP-mediated association with DP^84GGPM87^, or that an Ii region(s) other than CLIP is incompatible with the DP cleft, and may be responsible for this lack of association.

### CLIP encoded in full length Ii contributes to the lack of a CLIP-mediated association between DP^84GGPM87^ and Ii

**Figure 3:**
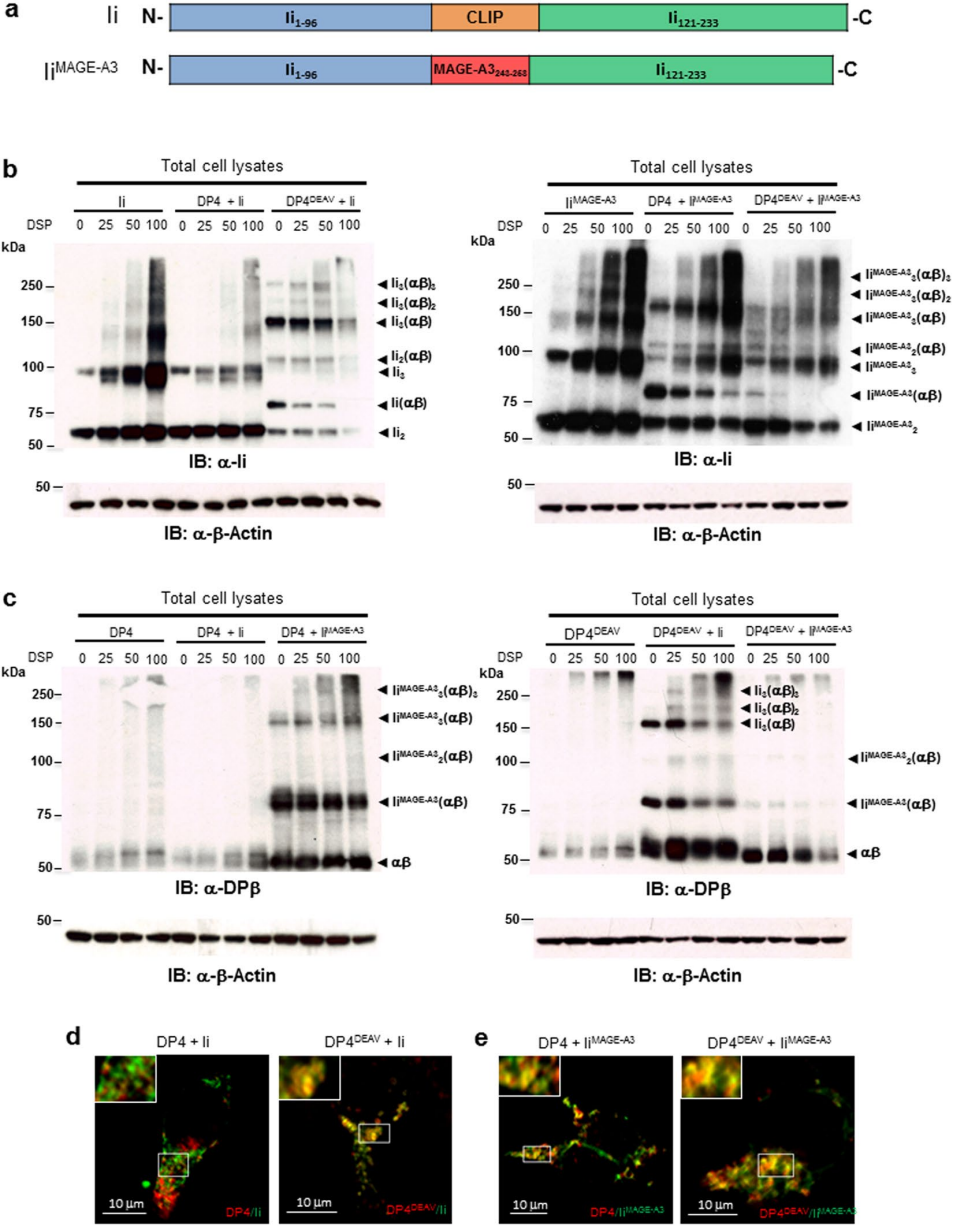
Substitution of the CLIP sequence with the DP4-restricted MAGE-A3_243–258_ peptide restores multimeric complex formation between Ii and DP^84GGPM87^. (**a**) Schema of chimeric Ii constructs encoding MAGE-A3_243–258_ peptide in place of Ii_97–120_ (CLIP) sequence. (**b,c**) HEK293 cells were transiently transfected with the indicated combination of genes and treated with the indicated concentration of the chemical crosslinker DSP for 2 hours. Non-reduced samples were immunoblotted with either anti-Ii (**b**) or anti-DPβ (**c**) mAb. Reduced samples were immunoblotted with anti-β-actin mAb. (**d,e**) HEK 293 cells were transiently transfected with either DP4 or DP4^84DEAV87^ as indicated and either Ii (**d**) or Ii^MAGE-A3^ (**e**). Cells were fixed, permeablized and stained using mAbs specific for HLA-DP (red) or Ii (green), then analyzed by confocal microscopy. Inset boxes depict areas shown at a higher magnification. Scale bar in all images represents 10 µm.

We next investigated which region(s) of Ii contribute to the lack of CLIP-region association with DP^84GGPM87^. In cells, Ii is proteolytically cleaved by endopeptidases including cathepsins at both N-and C-termini, leaving CLIP in the class II cleft. First, we tested whether the Ii-derived CLIP region itself does inhibit CLIP-region binding to DP^84GGPM87^. To this end, we generated a chimeric Ii encoding the DP4-restricted MAGE-A3_243–258_ peptide in place of CLIP (Fig. 3a). MAGE-A3 is an endogenous protein, whose MAGE-A3_243–258_ peptide is naturally processed and presented by DP4^16,21^. We previously showed that, unlike other class II, including DP^84DEAV87^, multimers were not formed between DP^84GGPM87^ and Ii due to the lack of CLIP region-mediated binding between them^16^. To study whether substitution of the CLIP region with another DP4-restricted peptide in Ii can restore multimer formation, lysates of DP4 or DP4^84DEAV87^–transduced HEK293 expressing either Ii or Ii^MAGE-A3^ were subjected to chemical cross-linking and subsequent western blotting analysis^16^. When blotted with anti-Ii (Fig. 3b) or anti-DPβ mAb (Fig. 3c), HEK293 cells transfected with wild-type Ii formed multimers with only DP4^84DEAV87^ but not wild type DP4 as previously shown^16^. However, Ii^MAGE-A3^ did generate multimers with both DP4 and DP4^84DEAV87^, though these multimers formed more readily with wild-type DP4. We have previously reported that CLIP-dependent binding between Ii and class II is linked to their co-localization as detected through confocal microscopy analysis^16^. Indeed, whereas wild-type Ii co-localized with only DP4^84DEAV87^ (Fig. 3d), Ii^MAGE-A3^ co-localized with both DP4^84DEAV87^ and wild-type DP4 (Fig. 3e). Together with the above observations that the CLIP peptide alone binds DP^84GGPM87^, these results indicate that the CLIP region contributes to the lack of a CLIP-mediated association between Ii and DP^84GGPM87^ only when it is encoded by full-length Ii, perhaps through the generation of an Ii conformation not amenable to CLIP binding.

### MAGE-A3_243–258_ peptide derived from chimeric Ii is efficiently processed and presented by DP4 regardless of Ii and HLA-DM

**Figure 4:**
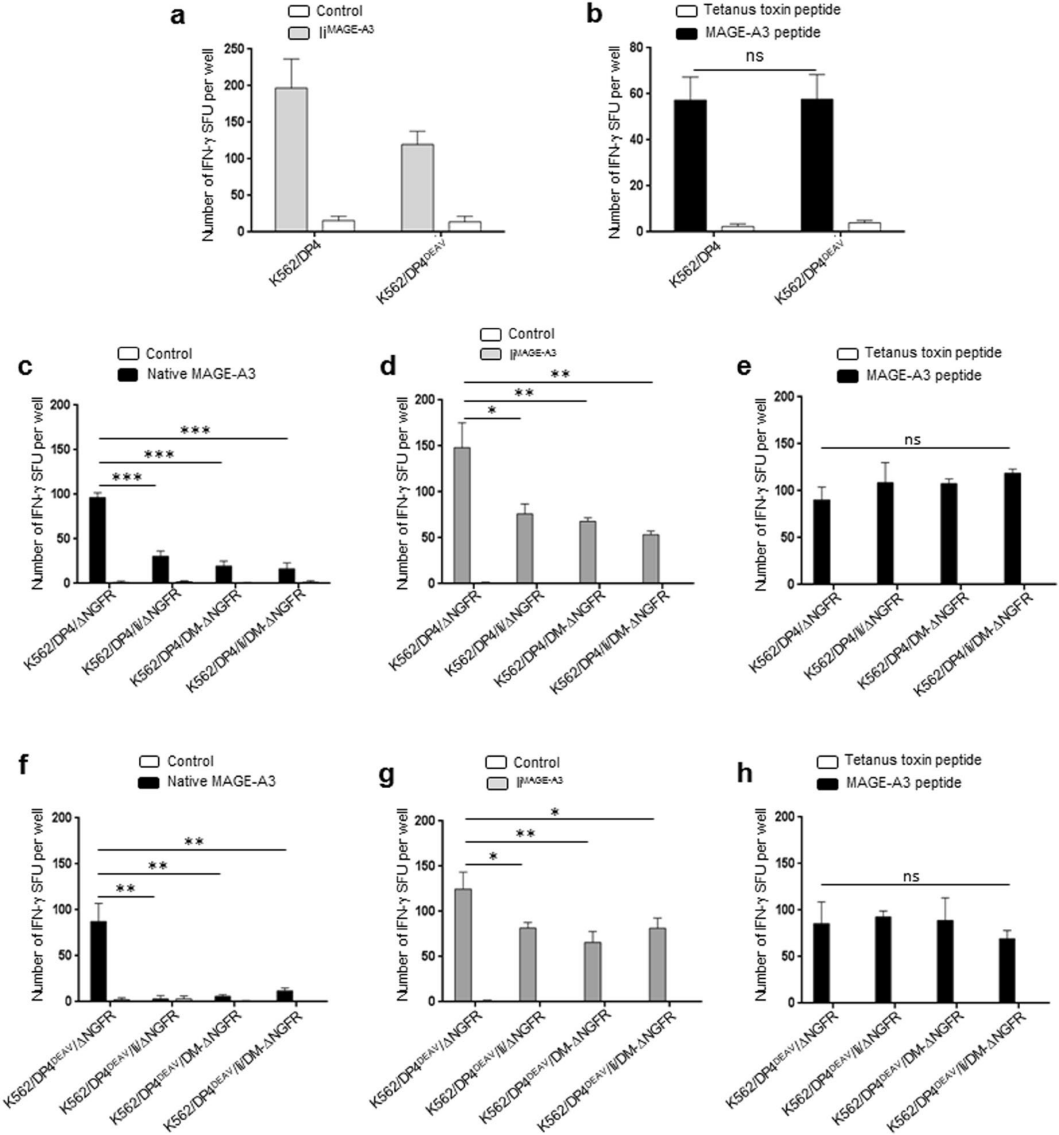
DP4 constitutively presents MAGE-A3_243–258_ peptide derived from chimeric Ii^MAGE-A3^ regardless of the presence of Ii and HLA-DM. (**a-h**) DP4/MAGE-A3-specific CD4^+^ T cells were stimulated using the indicated K562-based aAPCs, and IFN-γ responses were measured by ELISPOT analysis. (**a**) K562 cells stably expressing either DP4 or DP4^84DEAV87^ as indicated were transiently transfected with either Ii^MAGE-A3^ linked with IRES-EGFP, or empty vector (control) encoding IRES-EGFP alone, and used to stimulate T cells. (**b**) Immunogenicity of stimulator cells was evaluated by stimulating responder T cells with the indicated K562-based aAPCs following pulse with 10 µM of either Tetanus Toxin TT_947–967_ peptide (control) or MAGE-A3_243–258_ peptide. (**c,d**) K562 cells stably expressing DP4, either in the presence or absence of stably expressed Ii and/or HLA-DM as indicated, were transiently transfected with native MAGE-A3 (**c**), Ii^MAGE-A3^ (**d**), or empty vector (control) linked with IRES-EGFP, and used to stimulate T cells. (**e**) Immunogenicity of stimulator cells was evaluated by stimulating responder T cells with the indicated K562-based aAPCs following pulse with 10 µM of either Tetanus Toxin TT_947–967_ peptide (control) or MAGE-A3_243–258_ peptide. (**f,g**) K562 cells stably expressing DP4^84DEAV87^, either in the presence or absence of stably expressed Ii and/or HLA-DM as indicated, were transiently transfected with native MAGE-A3 (**f**), Ii^MAGE-A3^ (**g**), or empty vector (control) linked with IRES-EGFP and used to stimulate T cells. (**h**) Immunogenicity of stimulator cells was evaluated by stimulating responder T cells with the indicated K562-based aAPCs following pulse with 10 µM of either Tetanus Toxin TT_947–967_ peptide (control) or MAGE-A3_243–258_ peptide. Transient transfection efficiencies were normalized by EGFP expression as measured by flow cytometry (Supplementary Figure 3). The data shown represent the mean ± SD of experiments performed in triplicate. Results are representative of at least three independent experiments. ns: not significant; *p < 0.05, **p < 0.01, ***p < 0.001 by unpaired, two-tailed Welch’s t-test.

It has been reported that chimeric Ii can be used as a vector for the efficient delivery of tumor-associated antigens to class II for subsequent presentation to CD4^+^ T cells^22^. To confirm processing and presentation of MAGE_243–258_ peptide from chimeric Ii^MAGE-A3^ in our experimental system, K562 cells stably transduced with either DP4 or DP4^84DEAV87^ were transiently transfected with either Ii^MAGE-A3^ or empty vector (control). DP4/MAGE-A3_243–258_-specific TCR-transduced T cells were then stimulated with the K562 transfectants to measure specific T cell responses in IFN-γ ELISPOT assays. MAGE-A3_243–258_ derived from Ii^MAGE-A3^ was efficiently presented to the cell surface on both DP4 and DP4^84DEAV87^ (Fig. 4a). DP4 was more potent in inducing T cells responses than DP4^84DEAV87^, possibly because DP4 generated multimers with Ii^MAGE-A3^ more efficiently (Fig. 3b,c). When exogenously pulsed with the MAGE-A3_243–258_ peptide, these stimulator cells demonstrated similar abilities to evoke specific T cell responses (Fig. 4b).

We next compared the effect of class II-associated chaperones, Ii and HLA-DM, on the presentation of MAGE-A3_243–258_ peptide derived from full-length, native MAGE-A3 and Ii^MAGE-A3^ proteins. Ii^−^ and HLA-DM^−^ K562 cells, stably expressing DP4, in the presence or absence of stably transduced Ii, HLA-DM, or both chaperones, were transiently transfected with native MAGE-A3 (Fig. 4c), Ii^MAGE-A3^ (Fig. 4d) or empty vector (control). These cells were then used as stimulator cells in an IFN-γ ELISPOT assay to stimulate DP4/MAGE-A3_243–258_-specific T cells as described above. In the case of the MAGE-A3 antigens derived from both native MAGE-A3 and Ii^MAGE-A3^, both wild-type Ii and HLA-DM significantly abrogated the presentation of the MAGE-A3 peptide by DP4 (Fig. 4c,d). In the case of Ii, this may be due to the ability of Ii to facilitate the transport of class II along the endocytic pathway, away from the ER, where the MAGE-A3 antigen is presumably loaded onto receptive DP4 molecules^16^. In the case of HLA-DM, this finding is most likely due to the peptide editing function of DM; the MAGE-A3 peptide may not have sufficient affinity to resist editing by DM in favor of other peptides (Fig. 2b). When exogenously pulsed with the MAGE-A3_243–258_ peptide, these stimulator cells demonstrated similar abilities to evoke specific T cell responses (Fig. 4e).

We next examined the effect of Ii and HLA-DM on the presentation of both MAGE-A3 antigen sources on DP4^84DEAV87^. K562 cells stably transduced with DP4^84DEAV87^ in the presence or absence of stably transduced Ii, HLA-DM, or both chaperones, were transfected with full-length MAGE-A3 (Fig. 4f), Ii^MAGE-A3^ (Fig. 4g), or empty vector (control). Similarly to wild-type DP4, both Ii and HLA-DM abrogated the presentation of MAGE-A3 antigen derived from both MAGE-A3 sources. However, this decrease was more profound when native-MAGE-A3 was the antigen source, most likely due to both the modestly inferior ability of the MAGE-A3 peptide to associate with DP4^84DEAV87^ compared to DP4 (Fig. 2b), and the ability of full-length Ii to associate with DP4^84DEAV87^ via the CLIP region and block the binding of these antigens early in the endocytic pathway. When exogenously pulsed with the MAGE-A3_243–258_ peptide, these stimulator cells demonstrated similar abilities to evoke DP4/MAGE-A3_243–258_-specific T cell responses (Fig. 4h). In accordance with the observation that DP4 molecules seem to bind Ii^MAGE-A3^ via the MAGE-A3_243–258_ region in a similar manner as other class II molecules bind Ii via the CLIP region, these results demonstrate that the presentation of chimeric Ii^MAGE-A3^-derived MAGE-A3_243–258_ peptides on DP4 is efficient even in the presence of Ii and DM when compared to native, full-length MAGE-A3 as the source of antigen.

### N-terminal, but not C-terminal, non-CLIP Ii region(s) also contribute to the lack of CLIP-mediated DP^84GGPM87^-Ii association

**Figure 5:**
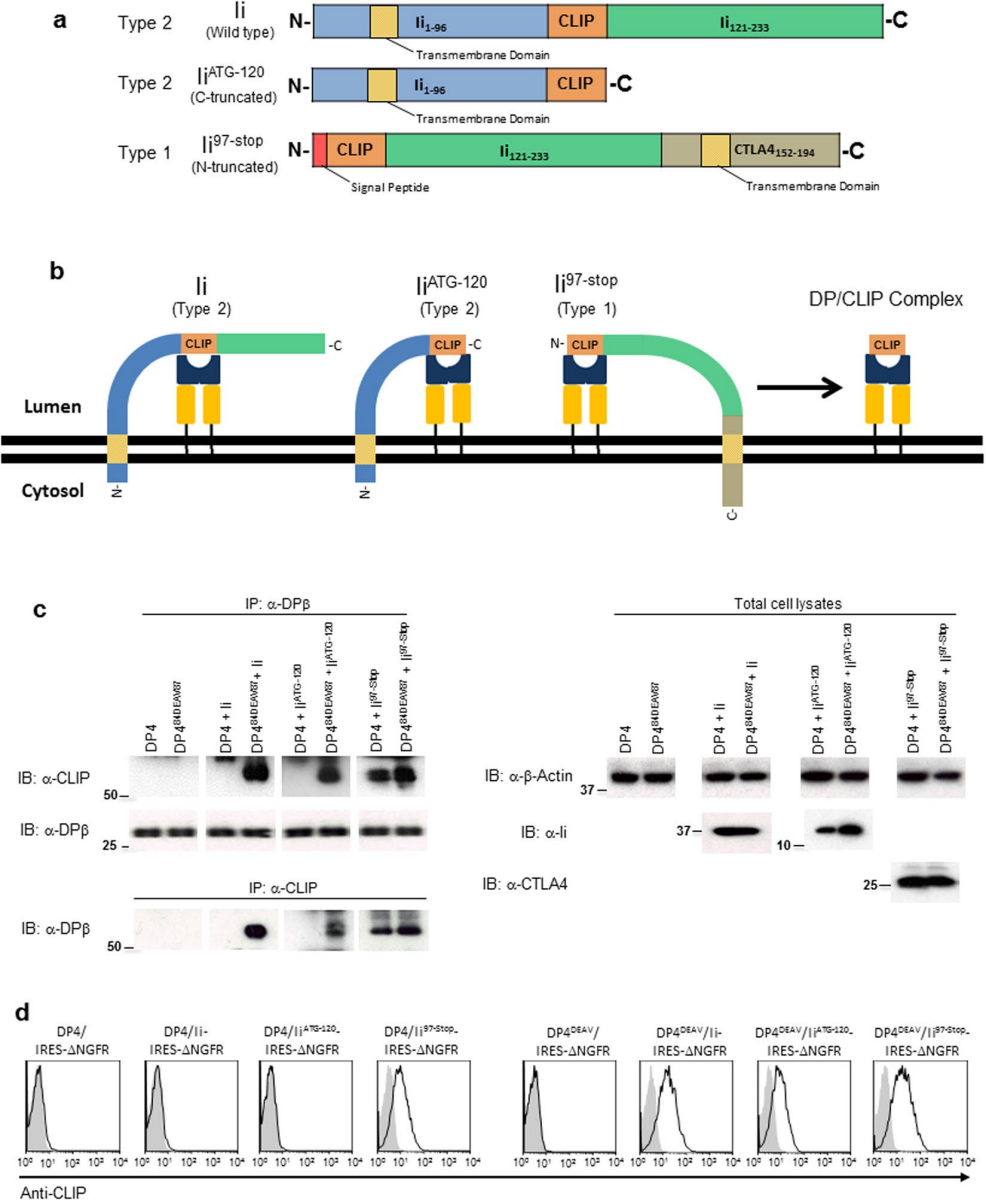
Truncation of N-terminal non-CLIP Ii region(s) restores CLIP-mediated Ii-DP association with DP4. (**a**) Schema depicting Ii C-terminal (Ii^ATG-120^) and N-terminal (Ii^97-Stop^) truncation mutants compared to wild-type Ii. Note that to generate Ii^97-Stop^, the transmembrane segment of CTLA4 was fused to the C-terminus of Ii to maintain membrane tethering. (**b**) Schema depicting Ii truncation mutants compared to wild-type Ii when membrane embedded. Addition of CTLA4 transmembrane region converts Ii^97-Stop^ to a type I transmembrane protein, and consequently the CLIP sequence in each construct is situated on the same side of the ER membrane allowing access to class II as depicted. (**c**) K562 cells stably expressing the indicated class II allele were transiently transfected with wild-type Ii, Ii^ATG-120^, Ii^97-Stop^ or empty vector (control). Lysates were then harvested from these cells, and non-reduced samples were immunoprecipitated and immunoblotted with the indicated mAbs. (**d**) K562 cells were stably transduced with the indicated combination of genes and then stained using anti-NGFR and anti-CLIP mAbs. The data shown are gated on ΔNGFR^+^ cells. Full, uncropped blots are provided in Supplementary Fig. 4.

To investigate whether a non-CLIP Ii region might also inhibit the association between DP^84GGPM87^ and Ii, we generated truncated Ii mutants lacking either N-terminal (Ii^97-Stop^) or C-terminal (Ii^ATG-120^) non-CLIP Ii region(s) (Fig. 5a). Since Ii is a type II transmembrane protein, a signal peptide and the CTLA4 transmembrane region were fused upstream and downstream of the N-terminally-truncated Ii, respectively, to generate membrane-tethered, type I Ii^97-Stop^. As depicted, this conversion allows class II molecules to access the CLIP sequence in each of these mutants in the same orientation, on the same side of the ER membrane (Fig. 5b). It is known that DP/CLIP complex formation is a consequence of successful CLIP-mediated Ii binding to DP^16^. To determine whether these mutant Ii molecules can bind to DP4 through their CLIP regions and produce such complexes, K562 cells were stably transduced with either DP4 or DP4^84DEAV87^ and transiently transfected with wild-type Ii, Ii^97-Stop^, Ii^ATG-120^ or empty vector (control). Lysates of these K562 cells were then immunoprecipitated and immunoblotted with anti-CLIP and anti-DPβ mAbs to detect DP/CLIP complex formation (Fig. 5c). As expected, all Ii constructs produced DP/CLIP complexes with DP4^84DEAV87^. However, only Ii^97-Stop^ generated such complexes with both wild-type DP4 and DP4^84DEAV87^; both wild-type Ii and Ii^ATG-120^ failed to yield wild-type DP4/CLIP complexes. To confirm these results, we performed flow cytometry experiments to investigate CLIP expression on the surface of K562 cells stably expressing either DP4 or DP4^84DEAV87^ and either Ii, Ii^ATG-120^, Ii^97-Stop^, or the empty vector linked to ΔNGFR. In accordance with our previous results, CLIP was only observed to bind to wild-type DP4 in the case of Ii^97-Stop^, whereas DP4^84DEAV87^ displayed the CLIP peptide derived from all forms of Ii (Fig. 5d). These results suggest that truncation of the N-terminal but not the C-terminal non-CLIP Ii region(s) is sufficient to restore DP/CLIP complex formation between Ii and DP4.

**Figure 6:**
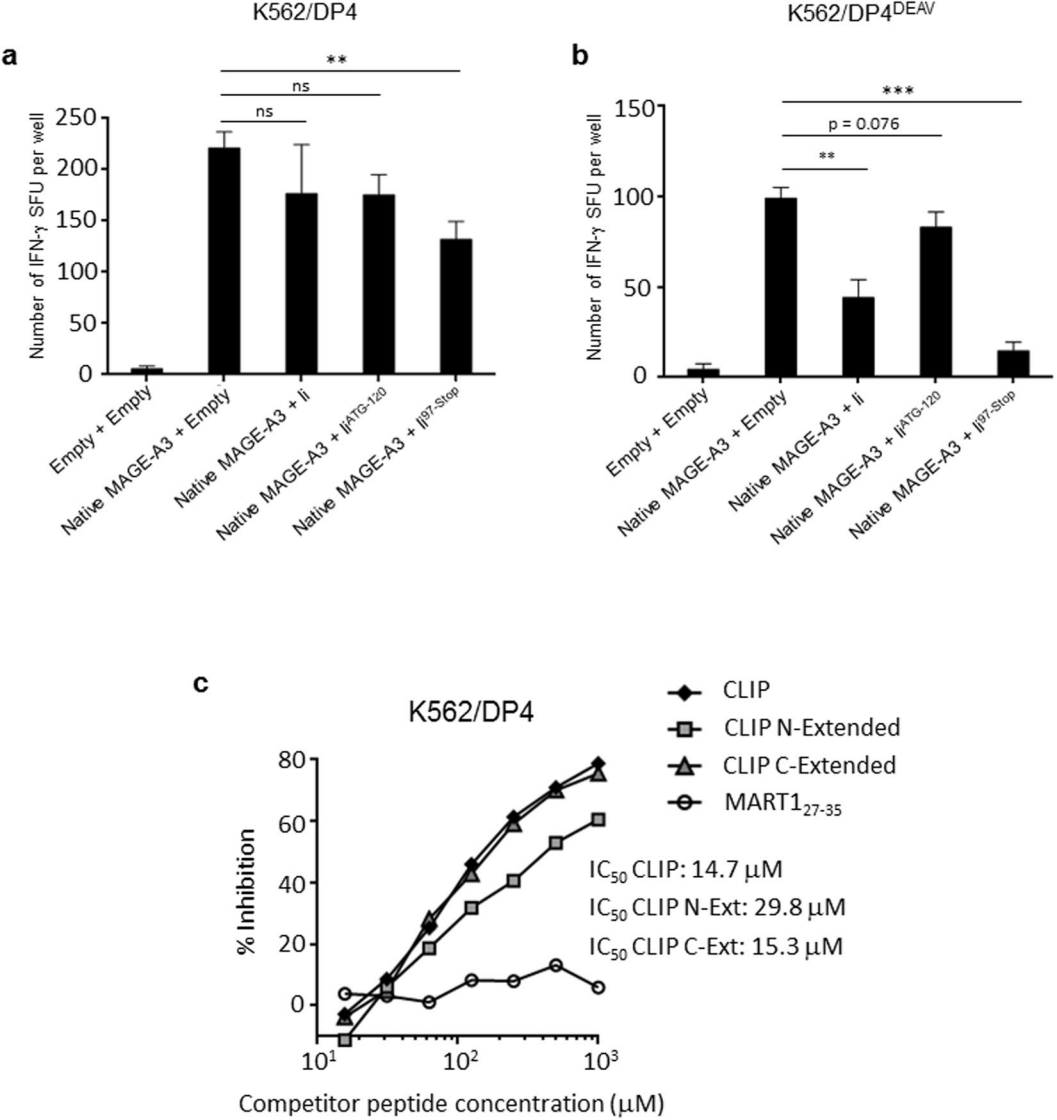
The N-terminal Ii region(s) inhibit CLIP-dependent binding to DP4 *in vivo* and *in vitro*. (**a,b**) DP4/MAGE-A3-specific CD4^+^ T cells were stimulated using the indicated K562-based aAPCs, and IFN-γ responses were measured by ELISPOT analysis. K562 cells stably expressing DP4 (**a**) or DP4^84DEAV87^ (**b**) were transiently transduced with either the empty vector or full-length native MAGE-A3, and one of wild-type Ii, Ii^ATG-120^, Ii^97-Stop^, or the empty vector as indicated and used to stimulate T cells. The transient transfection efficiencies were normalized to EGFP expression as measured by flow cytometry (Supplementary Figure 3). The data shown represent the mean ± SD of experiments performed in triplicate. The results are representative of at least three independent experiments. ns: not significant; **p < 0.01, ***p < 0.001 in unpaired, two-tailed Welch’s *t-*test. (**c**) A K562 cell-based competitive binding assay was performed using K562 cells stably expressing DP4. The cells were pulsed with graded concentrations of the CLIP, N-extended CLIP (by five amino acids), C-extended CLIP (by five amino acids), or MART1_27–35_ peptide in the presence of 2 μM of the biotin-conjugated reference peptide HIV ENV_31–45_. Then, the cells were stained with phycoerythrin-conjugated streptavidin and analyzed by flow cytometry. The percent inhibition of binding by the CLIP peptide was calculated as the mean fluorescence intensity (MFI) using the formula: %inhibition = [1 − (MFI with CLIP peptide/MFI without CLIP peptide)] × 100.

T cell activation assays were used to demonstrate the functional relevance of restored DP/CLIP complex formation between Ii^97-Stop^ and DP4. K562 cells were stably transduced with either DP4 or DP4^84DEAV87^ and either Ii, Ii^ATG-120^, Ii^97-Stop^ or empty vector. Then, cells were transiently transfected with the full-length native MAGE-A3 (or empty vector) and used as stimulator cells to measure specific T cell responses in IFN-γ ELISPOT assays. When presented on wild-type DP4, MAGE-A3 antigen presentation was only significantly impaired by Ii^97-Stop^; wild-type Ii and Ii^ATG-120^ were not able to abrogate this presentation (Fig. 6a). As expected, in the case of DP4^84DEAV87^, both wild-type Ii and Ii^97-Stop^ significantly reduced the presentation of MAGE-A3 (Fig. 6b); the reduction in MAGE-A3 presentation by Ii^ATG-120^ was not significant, most likely due to the absence of critical C-terminal non-CLIP binding regions, resulting in reduced DP-CLIP complex formation (Fig. 5c)^22^. Furthermore, in competitive peptide binding assays, C-extended CLIP peptide associated with DP4 at levels similar to that of the wild-type CLIP peptide, whereas N-extended CLIP peptide bound less effectively to DP4, supporting the conclusion that the N-terminal Ii region(s) contributed to the lack of CLIP-dependent association between Ii and DP4 (Fig. 6c). Taken together, these data suggest that the N-terminal Ii region(s) and the CLIP region itself come together to produce an unfavorable Ii conformation that does not allow the CLIP-mediated association of Ii with DP4.

## Discussion

Our previous work investigated the nature of the class II-Ii interaction from the class II perspective, and identified the critical polymorphism in the DPβ chain for determining CLIP-mediated binding of Ii to HLA-DP molecules^16^. The work presented here provides insights into the nature of this association from the Ii perspective, identifying the key regions of Ii responsible for inhibition of the DP-Ii interaction in class II molecules possessing the DP^84GGPM87^ phenotype. The finding that substitution of the CLIP region with DP^84GGPM87^-restricted peptides in Ii (chimeric Ii) can result in presentation of these peptides to the cell surface in the context of DP^84GGPM87^ is not inherently novel^23^. However, in conjunction with both crosslinking and confocal microscopy data, which indicate restoration of multimer formation between chimeric Ii and DP^84GGPM87^, these data suggest that modulating the conformation of the CLIP sequence can significantly impact the overall nature of the DP-Ii interaction, most likely through modulation of the total Ii conformation. This hypothesis is supported by several studies that report allele-specific differences in the interactions between other class II molecules and Ii^24–26^.

The surprising finding that synthetic CLIP peptides themselves can bind both DP4 and DP4^84DEAV87^ molecules comparably suggests that the CLIP peptide sequence alone is compatible with the DP4 cleft and that a non-CLIP region in Ii should also impact the DP4-Ii interaction as well. Indeed, we found that the absence of the N-terminal non-CLIP Ii region(s) restored CLIP-mediated binding of Ii to DP4. Furthermore, chimeric Ii^MAGE-A3^ which encodes the DP4-restricted MAGE-A3_243–258_ peptide in place of the CLIP sequence did form multimers with DP4 much like wild-type Ii do with other class II. The DP4-restricted MAGE-A3_243–258_ peptide was also highly efficiently processed and presented just as CLIP is by DP^84DEAV87^ in the absence of HLA-DM. This suggests that chimeric Ii encoding a different but similarly compatible DP^84GGPM87^–restricted peptide, such as MAGE-A3_243–258_, in lieu of CLIP can overcome the conformational constrictions of the N-terminal non-CLIP Ii region(s), allowing both multimer formation and efficient processing/expression of CLIP-region peptides to occur.

Curiously, the observed complete lack of CLIP region-dependent association with class II has only been reported in HLA-DP; to our knowledge, it has never been reported for HLA-DR or DQ alleles. The finding that the substitution of Asp with Gly, a much smaller amino acid, eliminates Ii’s ability to bind via CLIP to class II suggests that a conformational change occurs as a result of this polymorphism. One possibility could be the loss of intra-chain association(s) with basic residues that may act to “widen” the class II cleft, although no obvious basic residues with which such an association could be made are apparent. It is also possible that steric hindrance of Asp by the amino acids comprising the DPα chain may allow the cleft to exist in an “open” conformation to allow CLIP-mediated Ii binding. Another interesting observation is that, although no HLA-DR allele unable to associate with Ii via the CLIP region has been reported, many DR alleles possess the DRβ^84Gly^ phenotype despite being able to present CLIP on the cell surface^16^. This fact suggests that differences exist between DP and DR alleles that do not allow Gly at this position to interfere with CLIP binding. One possibility is that this inhibition may primarily be α-chain-mediated and that mutations of the β-chain may be able to “correct” for non-receptive α-chains by modulating β-chain conformation. Under this assumption, DRα chains may simply be more “permissive” for CLIP binding than DPα chains, and so these modulating polymorphisms to the β-chain are not required for DRα. If true, this would explain why only HLA-DP alleles have been reported to evade CLIP-mediated binding while still maintaining non-CLIP interactions with Ii. However, these ideas are conjectures at present.

The need to understand how different class II alleles interact with known components of the class II processing and presentation pathway is highlighted by our previous work and the clinical implications of DP alleles possessing the DP^84GGPM87^ phenotype. That a single amino acid polymorphism can so dramatically alter the repertoire of peptides available to be presented raises the intriguing question of the clinical relevance of this repertoire shift. Disease phenotypes associated with DP4 specifically have been largely absent in the past, with only a modest link to rheumatoid arthritis previously described^27^. However, recent studies have emerged that suggest links between DP^84GGPM87^ alleles and human disease. We and others have demonstrated a convincing association between the 84–87 region of the DPβ chain and the risk of anti-neutrophil cytoplasmic autoantibody-associated vasculitis, with alleles possessing the DP^84GGPM87^ genotype acting as risk alleles for the disease^28^. Importantly, both HLA–DPB1 and HLA–DPA1 were identified as significantly associated genes in the study, suggesting that it is likely that peptides presented by these DP^84GGPM87^ molecules contribute to disease pathogenesis. Furthermore, other groups have demonstrated a protective effect for DP^84GGPM87^ for chronic hepatitis B infection, with DP^84DEAV87^ existing as a risk allele for the disease^29,30^. Similarly, in a study of childhood acute lymphoblastic leukemia (ALL), individuals possessing DP^84GGPM87^, as opposed to DP^84DEAV87^, demonstrated enhanced event free survival^31^. Considering these associations, our previous and current studies may in fact suggest a mechanistic basis for these findings through the increased presentation of intracellular antigens on these class II alleles, ultimately promoting both an increased incidence of autoimmunity against intracellular antigens, as well as enhanced anti-viral and antitumor immunity in these patients. As the polymorphic nature of class II molecules continues to be implicated in various human diseases, deep understanding of the dynamic nature of the interactions between class II molecules and key components of its antigen processing and presentation pathway are critical for analyzing the dynamic repertoire of presented peptides, which can induce either pathogenic or protective CD4^+^ T cell responses depending on the context of human disease studied.

## Materials and Methods

### Reagents

Dithiobis(succinimidyl propionate) (DSP) was purchased from Sigma-Aldrich (St. Louis, MO). Peptides utilized in a surface binding assay study were CLIP (Ii_97–120_) (_97_LPKPPKPVSKMRMATPLLMQALPM_120_) and tetanus toxin_947–967_ (TT_947–967_) (_947_FNNFTVSFWLRVPKVSASHLE_967_). Peptides used in the competitive binding assay were HIV ENV_31–45_ (_31_ TEKLWVTVYYGVPVW_45_), MAGE-A3_243–258_ (_243_KKLLTQHFVQENYLEY_258_), N-extended CLIP (_92_NLRMKLPKPPKPVSKMRMATPLLMQALPM_120_), C-extended CLIP (_97_LPKPPKPVSKMRMATPLLMQALPMGALPQ_125_), and CLIP (as above). All peptides were purchased from GenScript (Piscataway, NJ).

### Cells, Culture and cDNA

Peripheral blood mononuclear cells (PBMCs) were collected from healthy donors after institutional review board approval. The University Health Network (UHN) Research Ethics Board (REB) approved this study, and all methods were performed in accordance with the relevant guidelines and regulations. Written, informed consent for both sample collection and study participation was obtained from every donor who provided these samples. K562 are human erythroleukemic cells which does not express MHC class I, MHC class II, Ii, nor HLA-DM. These cells were cultured in RPMI 1640 medium with the addition of 10% fetal calf serum (FCS) and gentamicin antibiotic (Life Technologies, Carlsbad, CA). HEK293 cells, which do not express class II, Ii, nor HLA-DM, were cultured in DMEM containing 10% FCS and gentamycin antibiotic (Life Technologies, Carlsbad, CA). All cell lines in this study were acquired from the American Type Culture Collection (ATCC) (Manassas, VA). Cell cultures were regularly monitored for the presence mycoplasma contamination using a Mycoplasma Detection Kit from the ATCC.

Stably generated K562-based cell lines were retrovirally transduced with cDNA encoding the indicated genes throughout the study. For both HLA-class II and Ii, successfully transduced cells were sorted using magnetic beads as previously described^18,19^. HLA-DP heterodimers in this study are always comprised of DPA1*01:03 (DPA1) paired with DPB1*04:01 (DPB4) to generate DP4, with substitution mutations made to the DPB4 chain as indicated. Several cDNA sequences were fused to a shortened version of the human nerve growth factor receptor (ΔNGFR) through an optimized Internal Ribosome Entry Site (IRES) sequence. This same sequence was used to link various cDNAs to EGFP for the sake of transient transfection efficiency monitoring. To generate appropriate T cells for T cell activation experiments, TCR α and β-chain cDNA specific for the DP4/MAGE-A3_243–258_ complex were generated based upon published sequences. All cDNA constructs described were cloned into the pMX backbone vector and had their sequence verified.

### Flow cytometry

mAbs specific for these surface/intracellular antigens were used in flow cytometry analyses: pan-HLA class II (6604366, 1:500, Beckman Coulter), invariant chain (Ii) (555540, 1:200, BD Biosciences & 357604, 1:200, BioLegend), CLIP (555981, 1:200, BD Biosciences), NGFR (557196, 1: 200, BD Biosciences) and HLA-DM (555983, 1: 200, BD Biosciences). Appropriate isotype controls were used at 1:500 or 1:200 dilutions as appropriate (BD Biosciences). Surface and intracellular flow cytometry staining protocols were performed as previously described^19,32^.

### Transient transfection

K562 and HEK293 cells were transfected with Lipofectamine 2000 transfection reagent (Life Technologies, Carlsbad, CA) and TransIT 293 (Mirus, Madison, WI), respectively, using the manufacturer’s protocols.

### *In vivo* chemical cross-linking

Intracellular crosslinking was performed using the chemical cross-linker Dithiobis(Succinimidyl Propionate) (DSP). Crosslinking solution was always freshly prepared as a 4 mg/ml working solution in dimethyl sulfoxide (DMSO) and diluted to the appropriate concentration in PBS for each experiment. Cells were cross-linked on ice for 2 hours, followed by incubation on ice for 10 min with quenching solution after removal of DSP. Quenching solution was discarded and cells were lysed in a pre-chilled 1% Nonidet P-40 extraction buffer.

### Confocal microscopy analyses

HEK293 cells were plated at a density of 5 × 10^4^ cells/well in a 6-well plate on coverslips, and transiently transfected at 60–70% confluency. 24 hours after transfection, cells were fixed and permeabilized with pure methanol pre-cooled at −20 °C for 12 min at room temperature. Cells were then incubated with the appropriate primary antibodies at 4 °C overnight, thoroughly washed with PBS, and incubated with the suitable secondary antibody at 37 °C for 1 hr. In cases where a conjugated primary antibody was used, coverslips were incubated with the conjugated antibody at 4 °C overnight. Coverslips were then washed 3 times with PBS and mounted with minimized quantities of Vectashield antifade reagent (Vector Labs, Burlingame, CA) to microscope slides. Primary and secondary antibodies included: mouse anti-Ii mAb (sc-6262, 1:200, Santa Cruz Biotechnology), anti-mouse conjugated with Alexa Fluor 488 (715-545-150, 1:1000, Jackson ImmunoResearch, West Grove, PA), and anti-HLA-DP mAb conjugated with DyLight594 (H1584, 1:200, Leinco Technologies, Inc., St. Louis, MO). A Zeiss LSM700 confocal microscope was used to capture fluorescence images.

### *In vitro* T cell activation assays

T cells obtained from healthy donors were purified using magnetic beads (Miltenyi Biotec) and then transduced with TCR genes specific for the DP4/MAGE-A3_243–258_ complex^33^. IFN-γ ELISPOT assays were performed as previously described^32,34,35^. Briefly, PVDF plates (Millipore, Bedford, MA) were incubated with capture mAb (1D1K; MABTECH, Mariemont, OH) at a 1:200 ratio overnight at 4 °C. After thorough washing with 2%FCS/PBS, DP4-MAGE-A3_243–258_-specific T cells were then incubated with 2 × 10^4^ of the indicated stimulator cells for 20–24 hours at 37 °C. After washing with PBS, plates were incubated with biotin-conjugated detection mAb (7-B6–1; MABTECH) at a 1:2000 ratio overnight at 4 °C. After washing with PBS, plates were incubated with HRP-conjugated Streptavidin (DAKO, Carpenteria, CA) at 1:5000, and IFN-γ spots were subsequently developed. The peptides used as controls were: MAGE-A3_243–258_ (_243_KKLLTQHFVQENYLEY_258_) (Genway, San Diego, CA) and tetanus toxin_947–967_ (_947_FNNFTVSFWLRVPKVSASHLE_967_) (Genway, San Diego, CA).

### Immunoprecipitation and western blotting

For both immunoprecipitation and western blotting experiments, cells were lysed in pre-chilled 1% Nonidet P-40 (NP-40) lysis buffer (20 mM Tris-HCl, pH 7.5, with 1 mM EDTA, 150 mM NaCl, 2.5 mM sodium pyrophosphate, 1 mM β-glycerophosphate, 1% NP-40, 1 μg/ml aprotinin and 1 mM PMSF). Cell lysates were spun at 12,000 rpm for 10 min at 4 °C, and if for the purpose of immunoprecipitation, were incubated overnight with 1 μg of mouse anti-DR/DP mAb (sc-51617, Santa Cruz Biotechnology) or 1μg of mouse anti-CLIP mAb (sc-12725, Santa Cruz Biotechnology) on 20 μl of either protein A Sepharose or protein G Sepharose (Santa Cruz Biotechnology, Santa Cruz, CA) beads, respectively, at 4 °C overnight with continuous tube turning. Beads were separated by centrifugation thoroughly washed with pre-chilled 1-% NP-40 lysis buffer. Immunoprecipitates/lysates were subjected to Tris-Glycine-based SDS-PAGE, and subsequent electrophoretic transfer to Immobilon-P membrane (Millipore, Bedford, MA). After blocking with 5% milk protein solution comprised of 0.1% Tween 20 in Tris-buffered saline, membranes were incubated with the appropriate primary antibody at 4 °C overnight. Membranes were then washed, and incubated with either HRP-conjugated goat anti-mouse IgG (H + L) secondary antibody (Promega, Madison, WI), or HRP-conjugated rat anti-mouse IgG VeriBlot secondary antibody (Abcam, Cambridge, MA), capable of detecting only non-denatured primary antibody to eliminate IP antibody signal, with continuous light shaking at room temperature for 1 hr. Images were collected using enhanced chemiluminescence (ECL) reagents (GE Healthcare) and photosensitive ECL films (GE Healthcare). Immunoprecipitating and primary antibodies used in western blotting experiments included: mouse anti-β-actin mouse mAb (sc-47778, 1:1500, Santa Cruz Biotechnology), mouse anti-CLIP mAb (sc-12725, 1:3000, Santa Cruz Biotechnology), mouse anti-DR/DP mAb (sc-51617, 1:2000, Santa Cruz Biotechnology), mouse anti-Ii mAb (sc-6262, 1:3000, Santa Cruz Biotechnology), and mouse anti-CTLA4 mAb (sc-376016, 1:1000, Santa Cruz Biotechnology). Uncropped blotting images are displayed in Supplementary Fig. 3.

### Statistical Analysis

Statistical analyses were carried out using GraphPad Prism 6.0. An unpaired, two-tailed Welch’s *t-*test was used for two-sample comparisons. *P* values of less than 0.05 were considered to be significant. All other numeric analyses described were also performed using GraphPad Prism 6.0. Sample size was not predetermined by any statistical method, nor were investigators blinded to any allocation procedures. No experiments were randomized.

### Data availability

Authors declare that all data supporting the conclusions of this study are provided either within the article and its related supplementary files, or are obtainable from authors on request.

## Electronic supplementary material

Supplementary Information
