# Supplementary material for: Mechanisms underlying the lack of endogenous processing and CLIP-mediated binding of the invariant chain by HLA-DP84Gly
Source: Sci Rep. 2018 Mar 19;8:4804. doi: 10.1038/s41598-018-22931-4 (PMC5859192; doi:10.1038/s41598-018-22931-4)
Supplement: Supplementary file 1 — Supplementary Information [file 41598_2018_22931_MOESM1_ESM.pdf]

Mechanisms underlying the lack of endogenous processing and CLIP-mediated binding of the invariant chain by HLA-DP^{84Gly}

Mark Anczurowski[1,2], Yuki Yamashita[1], Munehide Nakatsugawa[1], Toshiki Ochi[1], Yuki Kagoya[1], Tingxi Guo[1,2], Chung-Hsi Wang[1,2], Muhammed A. Rahman[1], Kayoko Saso[1], Marcus O. Butler[1,2,3], Naoto Hirano[*1,2]

[1]Tumor Immunotherapy Program, Campbell Family Institute for Breast Cancer Research, Campbell Family Cancer Research Institute, Princess Margaret Cancer Centre, University Health Network, Toronto, Ontario, Canada M5G 2M9 [2]Department of Immunology, University of Toronto, Toronto, Ontario, Canada M5S 1A8; [3]Department of Medicine, University of Toronto, Toronto, Ontario, Canada M5S 1A8

[*]Correspondence should be addressed to N.H.

Contact Information

Correspondence and requests for materials should be addressed to N.H. (naoto.hirano@uhnresearch.ca).

Naoto Hirano, MD, PhD

Princess Margaret Cancer Centre

610 University Avenue, Toronto, ON M5G 2M9, Canada

Phone: (416) 946-2190

Fax: (416) 946-6529

E-mail: naoto.hirano@uhnresearch.ca

Supplementary Figure 1

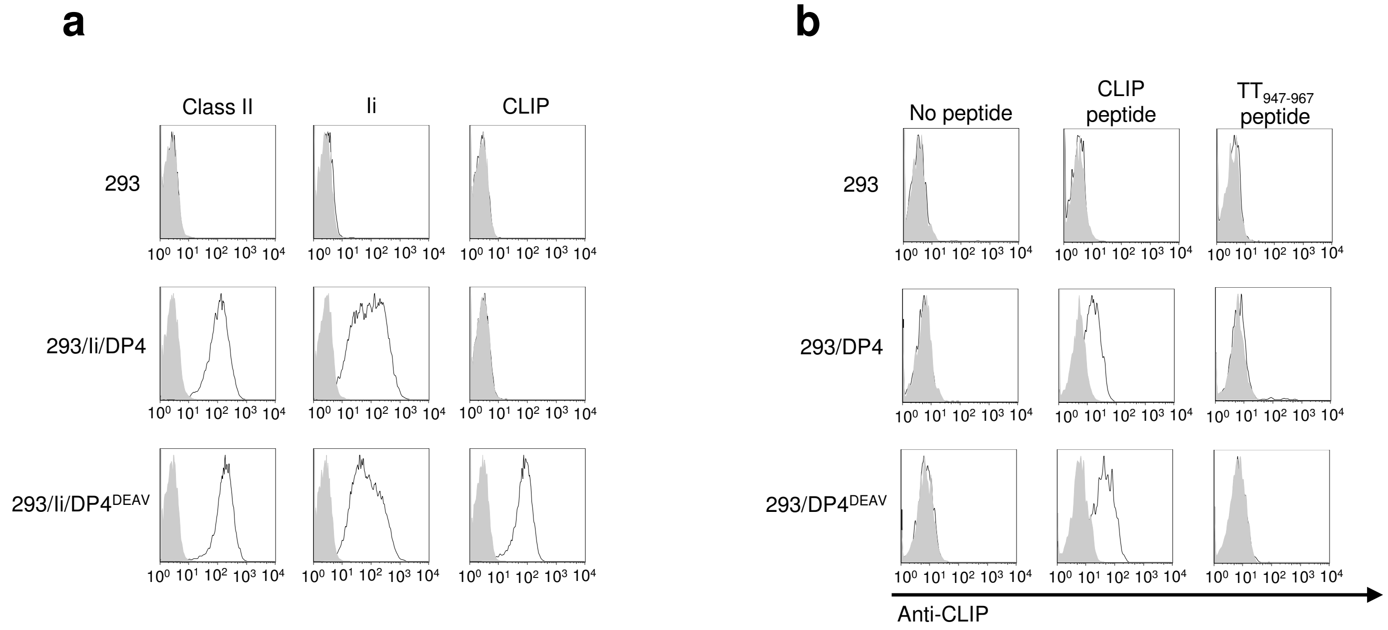

Supplementary Figure 1: CLIP derived from endogenous Ii is presented by HLA-DP4^{84DEAV87}, but not DP4, yet can associate with both DP4 and DP4^{84DEAV87}-expressing HEK293 cells when exogenously pulsed to the cell surface as a synthetic peptide. (**a**) Surface class II and CLIP expression, along with intracellular Ii expression, were analyzed on HEK293 transfectants by flow cytometry following staining with specific mAbs. HEK293 cells were retrovirally transduced with *DPA1*01:03* (DPA1) and either wild-type or mutated *DPB1*0401* (DPB4) with substitution mutations of the 84-87 region of the DPβ chain made as indicated. (**b**) Parental HEK293 or HEK293 cells stably expressing DP4 or DP4^{84DEAV87} were pulsed with 100 µM of CLIP, TT$_{947-967}$, or an equivalent volume of DMSO for 18 hrs. Surface CLIP expression was then analyzed by flow cytometry on the indicated HEK293 transfected cells following staining with CLIP-specific mAb.

Supplementary Figure 2

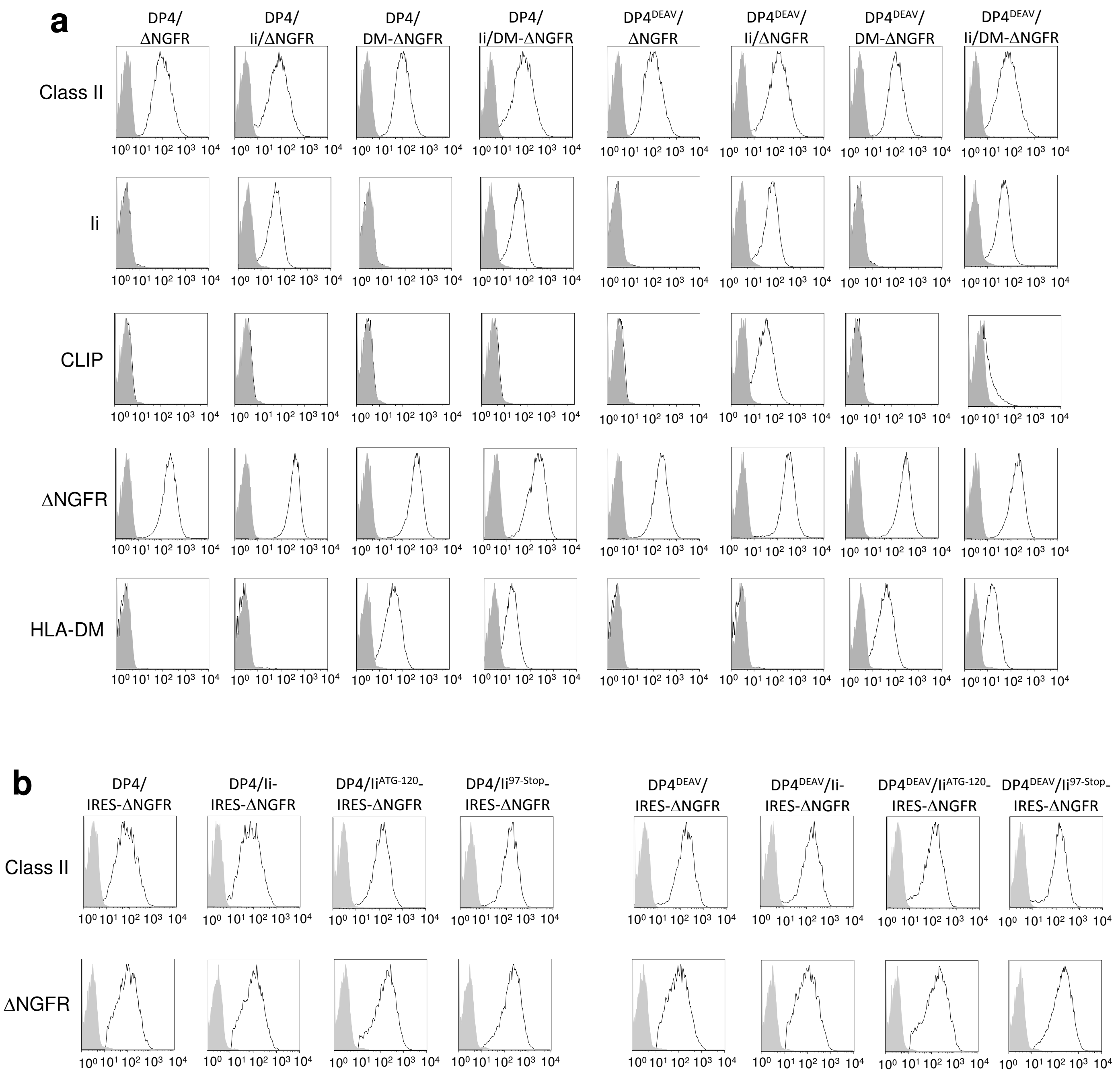

Supplementary Figure 2: Expression data for cells used in T cell activation (**Fig. 4**) (**a**) and flow cytometry experiments **(Fig. 5d) (b).** Surface class II, CLIP and ΔNGFR expression, along with intracellular Ii and HLA-DM expression as indicated, were analyzed on the indicated K562 transfectants by flow cytometry following staining with specific mAbs. K562 cells were retrovirally transduced with *DPA1*01:03* (DPA1) and either wild type or mutated *DPB1*0401* (DPB4) with substitution mutations of the 84-87 region of the DPβ chain made as indicated to generate DP4 or DP4^{84DEAV87} respectively.

Supplementary Figure 3

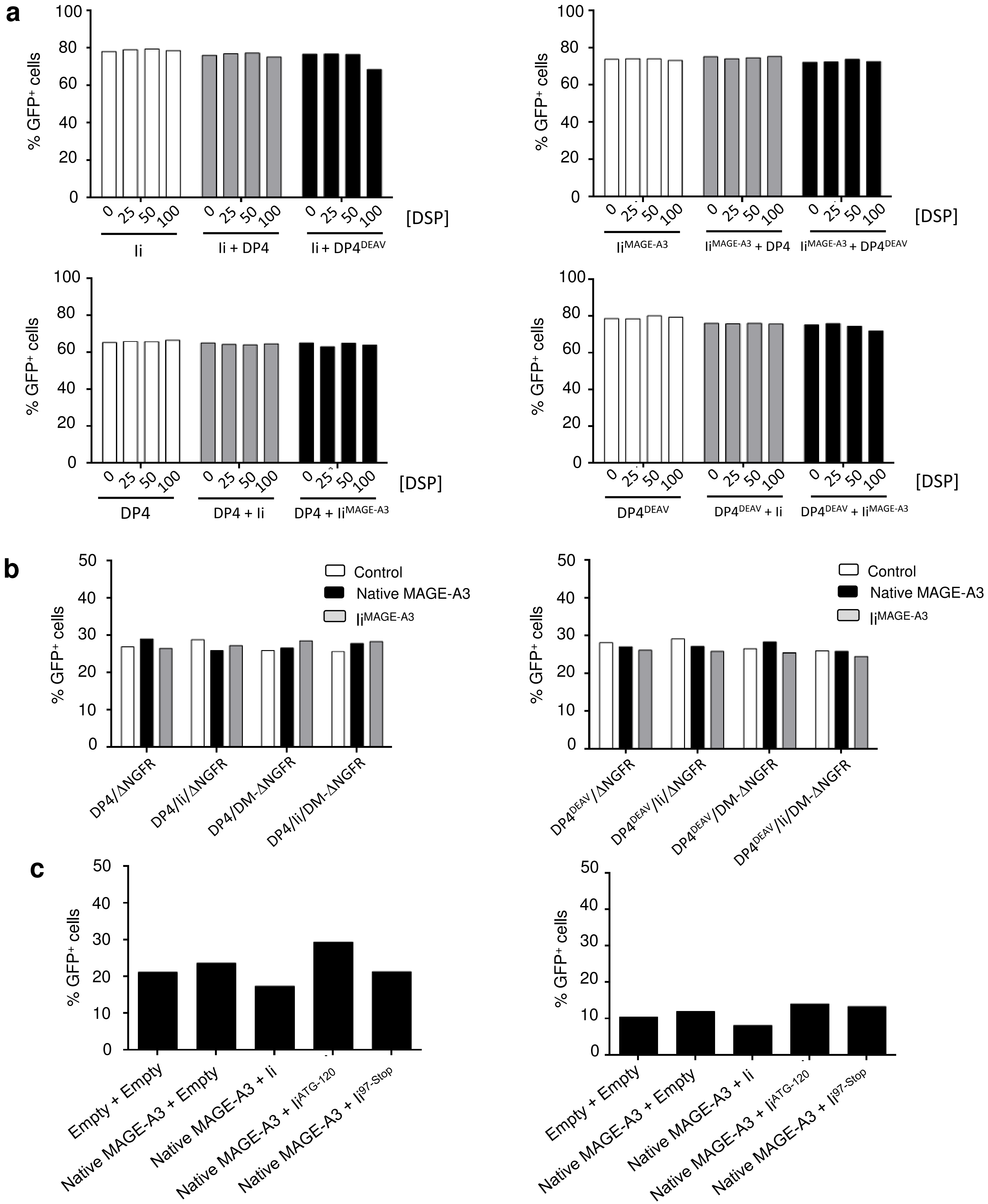

Supplementary Figure 3: Transfection efficiencies for cross-linked cells lysed in Figure 3b and 3c (**a**), as well as for stimulator cells used in ELISPOT analysis in Figure 4 (**b**) and Figure 6 (**c**). Transduction efficiencies are measured by GFP expression in cells by flow cytometry.

Supplementary Figure 4

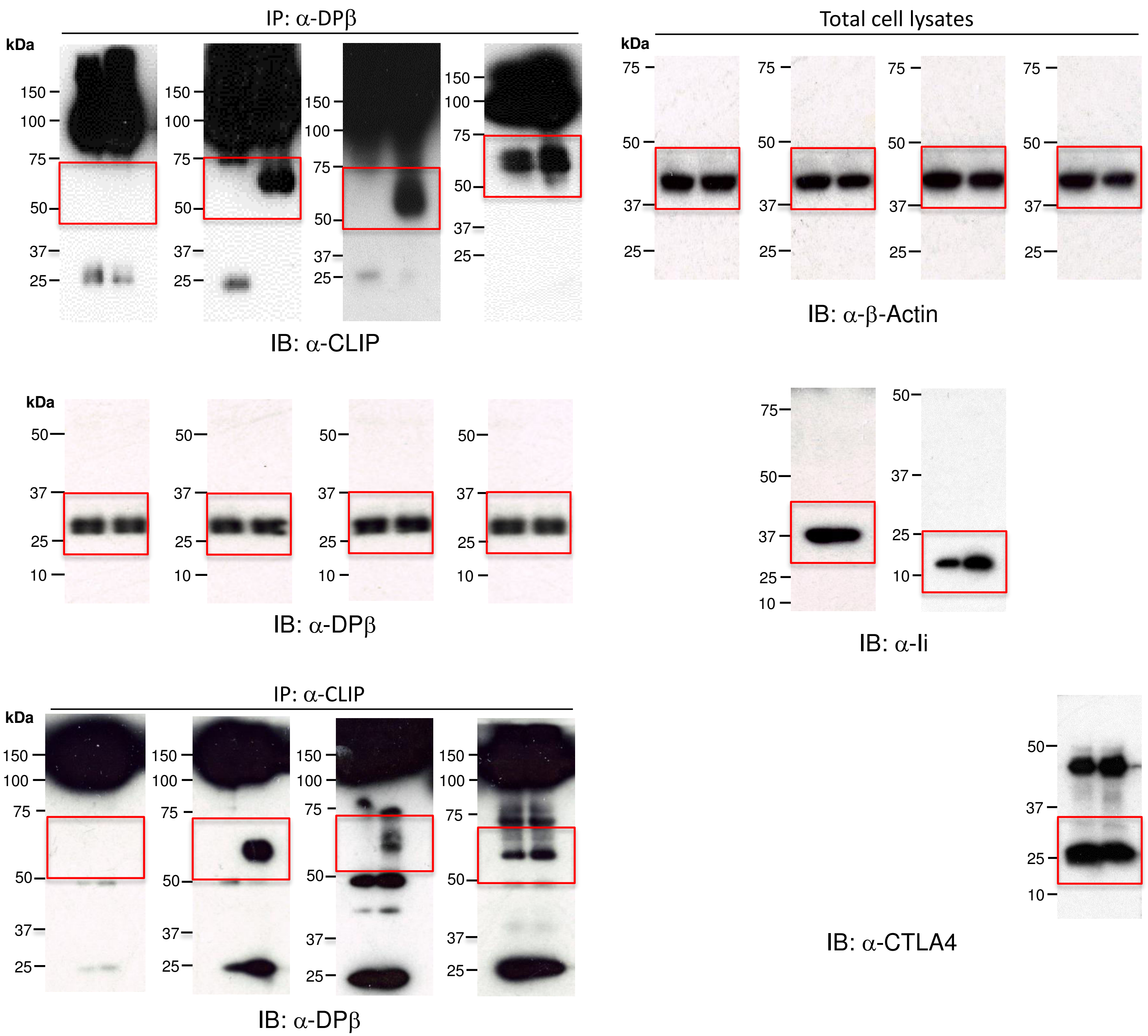

Supplementary Figure 4: Uncropped western blotting images from Figure 5c. Outlined regions represent portion of each image displayed in the main figures.
